# SNELM: SqueezeNet-Guided ELM for COVID-19 Recognition

**DOI:** 10.32604/csse.2023.034172

**Published:** 2023-01-20

**Authors:** Yudong Zhang, Muhammad Attique Khan, Ziquan Zhu, Shuihua Wang

**Affiliations:** 1 School of Computing and Mathematical Sciences, University of Leicester, Leicester, LE1 7RH, UK; 2 Department of Computer Science, HITEC University Taxila, Taxila, Pakistan

**Keywords:** SqueezeNet, complex bypass, transfer learning, extreme learning machine, COVID-19, deep learning, convolutional neural network, computed tomography

## Abstract

(Aim) The COVID-19 has caused 6.26 million deaths and 522.06 million confirmed cases till 17/May/2022. Chest computed tomography is a precise way to help clinicians diagnose COVID-19 patients. (Method) Two datasets are chosen for this study. The multiple-way data augmentation, including speckle noise, random translation, scaling, salt-and-pepper noise, vertical shear, Gamma correction, rotation, Gaussian noise, and horizontal shear, is harnessed to increase the size of the training set. Then, the SqueezeNet (SN) with complex bypass is used to generate SN features. Finally, the extreme learning machine (ELM) is used to serve as the classifier due to its simplicity of usage, quick learning speed, and great generalization performances. The number of hidden neurons in ELM is set to 2000. Ten runs of 10-fold cross-validation are implemented to generate impartial results. (Result) For the 296-image dataset, our SNELM model attains a sensitivity of 96.35 ± 1.50%, a specificity of 96.08 ± 1.05%, a precision of 96.10 ± 1.00%, and an accuracy of 96.22 ± 0.94%. For the 640-image dataset, the SNELM attains a sensitivity of 96.00 ± 1.25%, a specificity of 96.28 ± 1.16%, a precision of 96.28 ± 1.13%, and an accuracy of 96.14 ± 0.96%. (Conclusion) The proposed SNELM model is successful in diagnosing COVID-19. The performances of our model are higher than seven state-of-the-art COVID-19 recognition models.

## 1 Introduction

COVID-19 has caused 6.26 million deaths and 522.06 million confirmed cases till 17/May/2022. The polymerase chain reaction (PCR) can effectively detect its existence; however, the cluster of false-positive [1] perplexes clinicians. The chest computed tomography (CCT) [2] is another precise way to help clinicians to diagnose COVID-19 patients. Till July/2022, three vaccines are approved for use in UK, including Moderna, Oxford/AstraZeneca, and Pfizer/BioNTech.

In the recent few years, scholars proposed to novel artificial intelligence (AI)-based models for COVID-19 diagnosis. For examples, El-kenawy et al. [3] proposed an innovative feature selection and voting (FSV) classifier Wu [4] proposed a three-segment biogeography-based optimization (3SBBO) method for COVID-19 detection. Zhang [5] proposed a model combining a convolutional neural network (CNN) with stochastic pooling (SP). Their method is renamed CNNSP. Chen [6] merged gray-level co-occurrence matrix (GCM) and support vector machine (SVM) for COVID-19 classification. This method is named GCMSVM. Wang [7] proposed a wavelet entropy and Jaya (WEJ) algorithm. Pi [8] merged GCM with Schmitt neural network (SNN) for COVID-19 diagnosis. Their model is named GCMSNN. Wang [9] introduced self-adaptive particle swarm optimization (SaPSO) for COVID-19 detection. Ni et al. [10] proposed a deep learning approach (DLA) to characterize COVID-19. Wang et al. [11] developed a weakly supervised framework. Their model was named DeCovNet. Gafoor et al. [12] developed a deep learning model (DLM) to detect COVID-19 using chest X-ray.

Nevertheless, the above models still have room to improve in terms of their recognition performances, i.e., the accuracy. Inspired by the model in Özyurt et al. [13], we proposed SqueezeNet-guided ELM (SNELM), which combines traditional SqueezeNet (SN) with the extreme learning machine (ELM). Nevertheless, our SNELM is different from [13] in two ways. First, we do not use fuzzy C-means for super-resolution. Second, we choose the SN model with complex bypass, while [13] chooses the vanilla SN model. Our experiments show the effectiveness of this proposed SNELM model. In all, this study has several novel contributions:

(a) The multiple-way data augmentation (MDA) is used to increase the size of the training set.
(b) We propose the novel SNELM model to diagnose COVID-19.
(c) SNELM model gives higher results than seven state-of-the-art models.

## 2 Dataset and Preprocessing

Two datasets (D1 and D2) are used since they can report the results more unbiasedly. The details of the two datasets can be found in [4,5]. Table 1 displays the descriptions of D1 and D2. Suppose *n*_1_ stands for the number of subjects, and *n_2_* the number of CCT images. It is easy to observe that there are *n*_2_ = 296 images in D1 and *n*_2_ = 640 images in D2.

**Figure 1:**
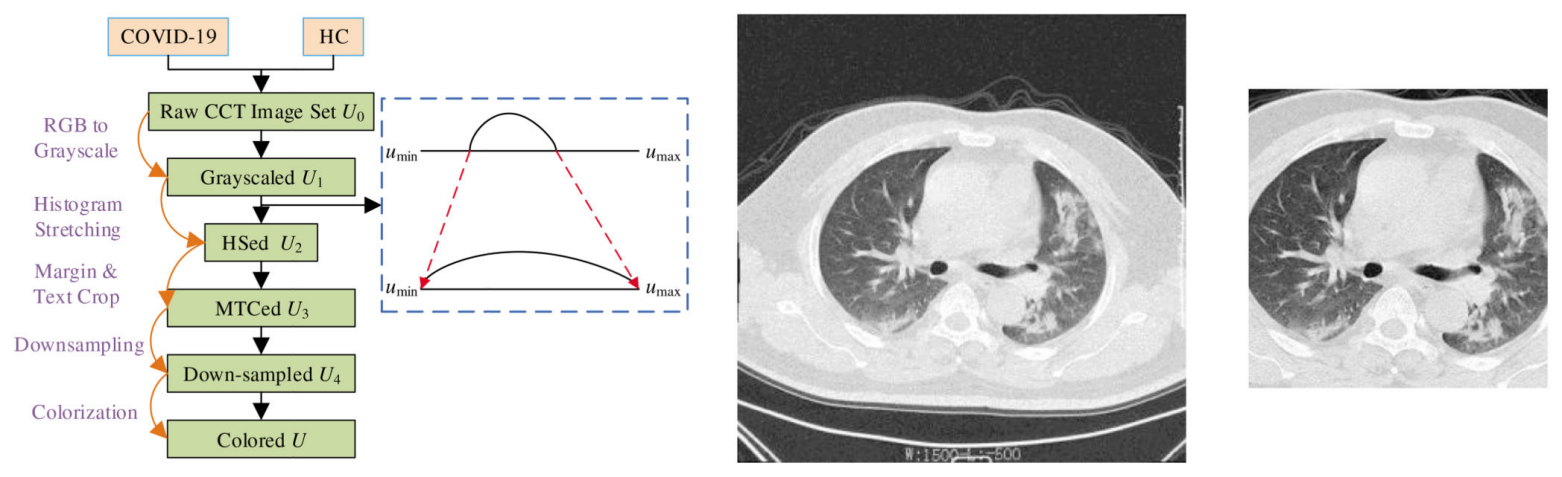
Preprocessing

A five-step preprocessing is employed. The flowchart can be seen in Fig. 1a, in which the five steps are grayscaling, histogram stretching (HS), margin and text crop (MTC), downsampling (DS), and coloriazation. Here *U* stands for the dataset at each step. HS is used to enhance the contrast. Suppose *U*_1_ = {*u*_1_(*k*)}, we first need to calculate its lower bound $u_{1}^{L}(k)$ and upper bound $u_{1}^{U}(k)$ as:

(1) $$\left\{ \begin{array}{l} u_{1}^{U}(k)=\underset{x}{\max}\underset{y}{\max}u_{1}(x,y|k)\\ u_{1}^{L}(k)=\underset{x}{\min}\underset{y}{\min}u_{1}(x,y|k) \end{array}\right.,$$

and the HSed image is defined as

(2) $$u_{2}(k)=\frac{u_{1}(k)-u_{1}^{L}(k)}{u_{1}^{U}(k)-u_{1}^{L}(k)}.$$

The grayscale range of *u*_2_(*k*) is [*u*_min_, *u*_max_]. Figs. 1b and 1c show the raw COVID-19 and preprocessed images, respectively. The downsampled dataset is symbolized as *U*_4_ = {*u*_4_(*k*)} with the size of each image as (*a*_1_, *a*_2_). The final grayscale image *u*_4_(*k*) is then stacked along channel direction to output the color image *u*(*k*):

(3) $$u(k)=f_{cat}^{channel}[u_{4}(k),u_{4}(k),u_{4}(k)],$$

where $f_{cat}^{channel}$ denotes the catenation function along the channel direction. The size of *u*(*k*) is now *a*_1_ × *a*_2_ × 3.

## 3 Description of SNELM

### 3.1 Multiple-Way Data Augmentation

**Figure 2:**
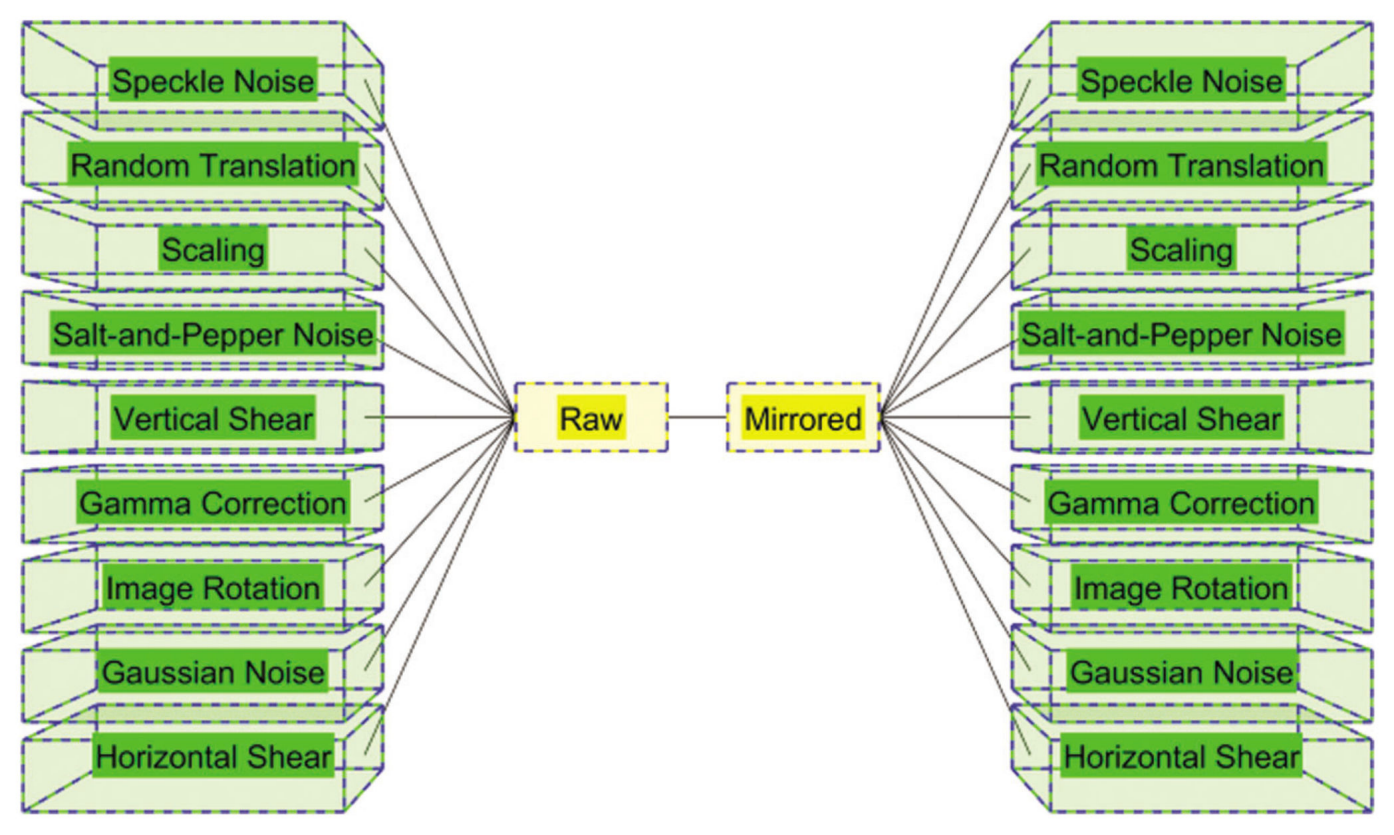
Schematic of MDA

Table 2 itemizes the abbreviation and their meanings. Fig. 2 illustrates the schematic of MDA. Assume the original image is *u*(*k*), then the horizontally mirrored image (HMI) is defined as *u^HMI^*(*k*) as

(4) $$u^{HMI}(x,y|k)=u(a_{1}-x,y|k),$$

where we do not take color channels into consideration. Then, all the *b*_1_ different data augmentation (DA) methods $g_{i}^{DA},i=1,\ldots ,b_{1}$ are applied to both *u*(*k*) and *u^HMI^*(*k*). Suppose each DA generates *b*_2_ new images. Finally, the whole generated images Λ(*k*) are defined as:

(5) $$u(k)\mapsto \Lambda (k)=f_{con}^{image}\left\{\begin{array}{cc} u(k) & u^{HMI}(k)\\ \underset{b_{2}}{\underset{︸}{g_{\left(1\right)}^{DA}[u(k)]}} & \underset{b_{2}}{\underset{︸}{g_{\left(1\right)}^{DA}[u^{HMI}(k)]}}\\ \cdots & \cdots \\ \underset{b_{2}}{\underset{︸}{g_{\left(b_{1}\right)}^{DA}[u(k)]}} & \underset{b_{2}}{\underset{︸}{g_{\left(b_{1}\right)}^{DA}[u^{HMI}(k)]}} \end{array}\right\},$$

where $f_{con}^{image}$ is the concatenation function along the image direction. The augmentation factor of MDA (AFMDA) is defined as:

(6) $$b_{3}=\frac{\left|\Lambda (k)\right|}{u(k)}=2\times b_{1}\times b_{2}+2.$$

Compared to normal individual DA methods, the MDA fuse the separate DA methods together and thus can yield better performances [14].

### 3.2 Fire Module and SqueezeNet with Complex Bypass

SqueezeNet (SN) is chosen since it can achieve a 50× reduction in model size compared to AlexNet and maintain the same accuracy [15]. This lightweight SN can help make our final COVID-19 recognition model fast and still have sufficient accuracy.

**Figure 3:**
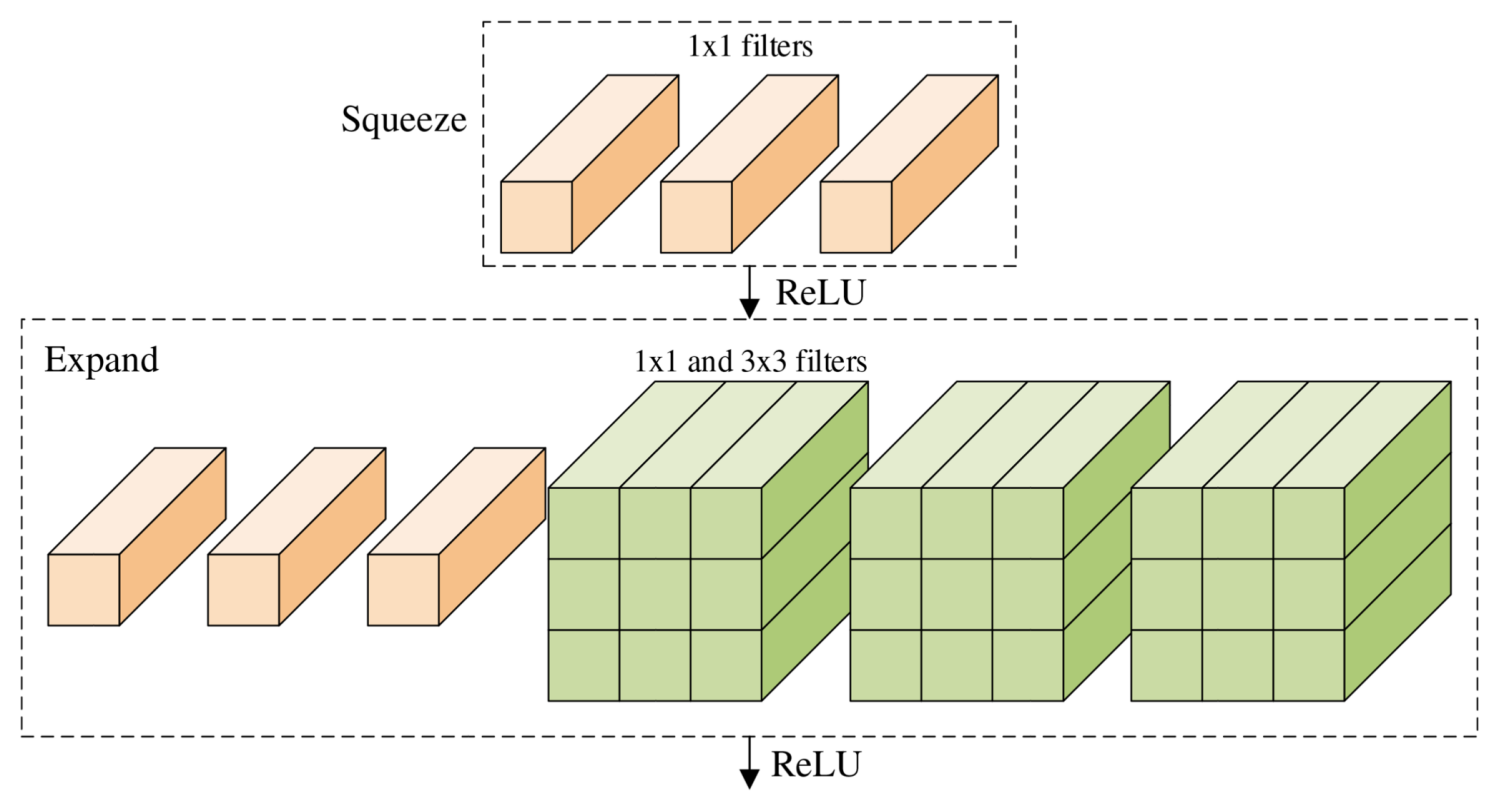
Structure of FM (*s*_1×1_ = 3, *e*_1×1_ = 3, e_3×3_ = 3)

The fire module (FM) is the core component in the N. It contains a squeeze layer (SL), which uses only 1 × 1 kernels, followed by an expand layer (EL), which contains several 1 × 1 and 3 × 3 kernels [16]. The structure of FM is shown in Fig. 3. Three tunable hyperparameters need to be tuned in an FM: *s*_1×1_, *e*_1×1_, and *e*_3×3_, which stand for the number of 1 × 1 kernels in the SL, and the number of 1 × 1 and 3 × 3 kernels in the EL.

Compared to ordinary convolutional neural network (CNN) architectures, the SN [17] has three main advantages: (i) replace traditional 3 × 3 kernels with 1 × 1 kernels. (ii) drop the number of input channels to 3 × 3 kernels using SLs. (iii) downsample late in SN, so the convolution layers have large activation maps [18].

**Figure 4:**
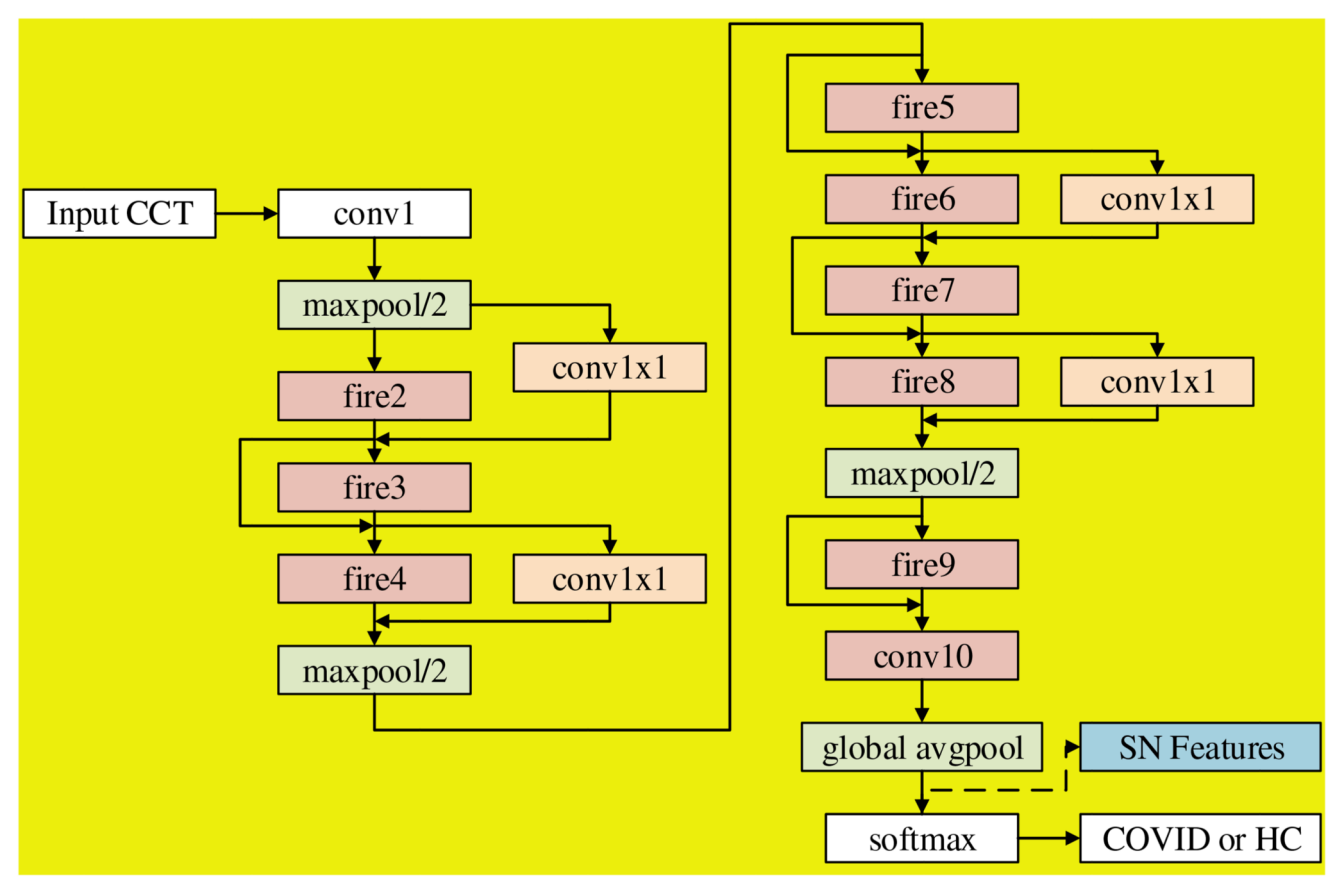
Flowchart of SN with simple bypass and complex bypass

There are different variants of SN. Özyurt et al. [13] used vanilla SN, while our SNELM use SN with complex bypass. Fig. 4 shows the flowchart, where we can observe not only simple bypass but also complex bypass are added between some FMs. If the “same-number-of-channel” requirement is met, a simple bypass is added. If that requirement is not met, a complex bypass is added. These bypasses can help improve the recognition performances, and their designs are similar to those in ResNet.

### 3.3 SN-Guided ELM

**Figure 5:**
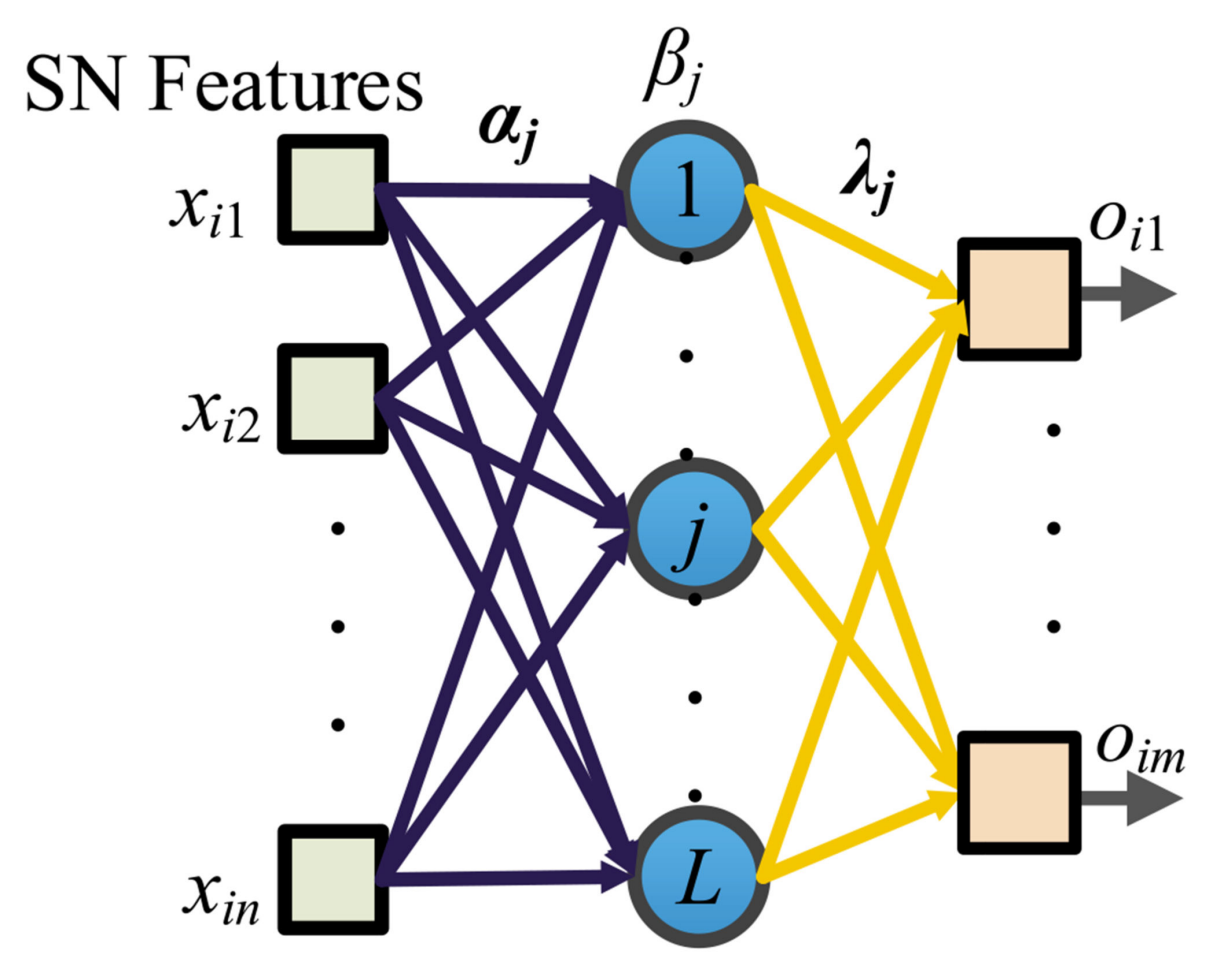
Schematic of SN-guided ELM

The SN features after global avgpool (See Fig. 4) are used as the learnt features and passed to the extreme learning machine (ELM) [19] that features a very fast classifier. Besides, ELM is simple to use, has greater generalization performance, and is appropriate for several nonlinear kernel functions and activation functions. Its structure is a single hidden-layer feedforward network shown in Fig. 5.

Let the *i*-th input sample be ***x***_*i*_ = (*x*_*i*1_, …, *x*_*in*_)^T^ ∈ R^*n*^; *i* = 1, …, *N*. The output of an ELM with *L* hidden neurons is:

(7) $$O_{i}=\sum_{j=1}^{L}\lambda_{j}h(\alpha_{j}x_{i}+\beta_{j}),i=1,\ldots ,N,$$

where *h* stands for the activation function, *α_j_* = (*α*_*j*1_, *α*_*j*2_, …, *α_jn_*)^T^ the input weight, *β_j_* the bias, *O_i_* = (*o*_*i*1_, *o*_*i*2_, *o*_*i*3_, …*o_im_*)^T^ the output of the model for the *i*-th input sample. Afterwards, the model is trained to yield

(8) $$\sum_{j=1}^{L}\lambda_{j}h(\alpha_{j}x_{i}+\beta_{j})=y_{i},\;i=1,\ldots ,N.$$

Let us rephrase the above equation as

(9) Mλ=Y,

where

(10) $$M(\alpha_{1},\ldots ,\alpha_{L},\beta_{1},\ldots ,\beta_{L},x_{1},\ldots ,x_{N})={\left[\begin{array}{ccc} h(\alpha_{1}x_{1}+\beta_{1}) & \cdots & h(\alpha_{L}x_{1}+\beta_{L})\\ \vdots & \ddots & \vdots \\ h(\alpha_{1}x_{N}+\beta_{1}) & \cdots & h(\alpha_{L}x_{N}+\beta_{L}) \end{array}\right]}_{N\times L},$$

(11) $$\lambda ={\left[\begin{array}{c} \lambda_{1}^{T}\\ \vdots \\ \lambda_{L}^{T} \end{array}\right]}_{L\times m},Y={\left[\begin{array}{c} y_{1}^{T}\\ \vdots \\ y_{L}^{T} \end{array}\right]}_{N\times m}.$$

It challenges the users to acquire the optimal ***α***_*j*_, *β_j_* and *λ_j_*. ELM can yield a solution quickly via the pseudo inverse:

(12) $$\lambda =M^{†}Y\text{,}$$

where ***M***^†^ signifies the Moore-Penrose [20]of ***M***. The pseudocode is shown in Algorithm 1.

**Algorithm 1: ELM**

Input SN features [***x***_*i*_, ***y***_*i*_].

Step A Initialize values of input weight ***α***_*j*_ and the bias *β_j_* randomly.

Step B Compute the output matrix ***M*** using Eq. (10).

Step C Compute the output weight *λ* using the pseudo inverse in (12). Output The trained ELM model.

### 3.4 Cross-Validation and Evaluation

**Figure 6:**
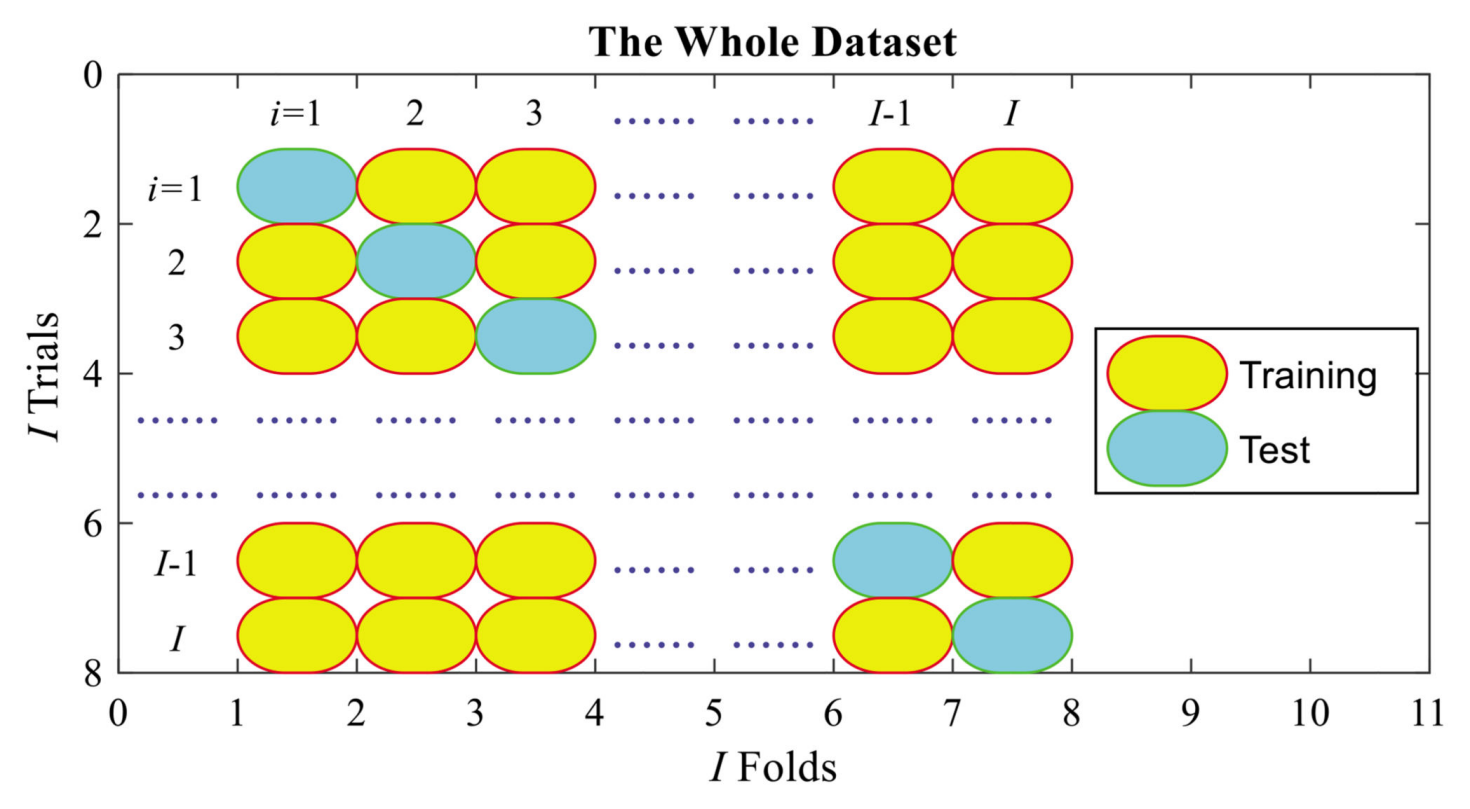
Schematic of one run of ***I***-fold CV

*T* runs of *I*-fold cross-validation (CV) are carried out. Assume the test confusion matrix (TCM, symbolized as Θ) over *t*-th run and *i*-th fold is:

(13) $$\text{Θ}(t,i)=\left[\begin{array}{cc} \theta_{11}(t,i) & \theta_{12}(t,i)\\ \theta_{21}(t,i) & \theta_{22}(t,i) \end{array}\right],$$

where *i* = 1, …, *I* stands for the fold index, and *t* = 1, …, *T* the run index. The (*θ*_11_, *θ*_12_, *θ*_21_, *θ*_22_) signify true positive, false negative, false positive, and true negative, respectively. At *i*-th trial, the *i*-th fold is employed as test, and the left folds {1, …, *i* – 1, *i* + 1, …, *I*} altogether are employed as training, as shown in Fig. 6, here one *I*-fold CV consists of *I* trials.

Θ(*t, i*) is gauged based on the *i*-th fold, which is the test set. We afterward take their summation across altogether *I* trials, as shown in Fig. 6. The TCM at *t*-th run Θ(*t*) is attained as

(14) $$\text{Θ}(t)=\sum_{i=1}^{I}\text{Θ}(t,i).$$

At *t*-th run, seven indicators $\vec{\kappa }(t)$ based on the TCM are calculated and concatenated in a whole as Θ(*t*):

(15) $$\text{Θ}(t)\mapsto \vec{\kappa }(t)=\{\kappa_{m}(t),m=1,\ldots ,7\},$$

where the first four indicators mean: κ_1_ sensitivity, *κ*_2_ specificity, *κ*_3_ precision, and *κ*_4_ accuracy as:

(16) $$\{\begin{array}{c} \kappa_{1}(t)=\frac{\theta_{11}(t)}{\theta_{11}(t)+\theta_{12}(t)}\\ \kappa_{2}(t)=\frac{\theta_{22}(t)}{\theta_{22}(t)+\theta_{21}(t)}\\ \kappa_{3}(t)=\frac{\theta_{11}(t)}{\theta_{11}(t)+\theta_{21}(t)}\\ \kappa_{4}(t)=\frac{\theta_{11}(t)+\theta_{22}(t)}{\theta_{11}(t)+\theta_{12}(t)+\theta_{21}(t)+\theta_{22}(t)} \end{array}.$$

*κ*_5_ is F1 score:

(17) $$\kappa_{5}(t)=2\times \frac{\kappa_{3}(t)\times \kappa_{1}(t)}{\kappa_{3}(t)+\kappa_{1}(t)}=\frac{2\times \theta_{11}(t)}{2\times \theta_{11}(t)+\theta_{12}(t)+\theta_{21}(t)},$$

*κ*_6_ is Matthews correlation coefficient (MCC), which is a more reliable statistical rate that produces a high score only if the prediction obtained good results in all of the four entries in the TCM [21].

(18) $$\kappa_{6}(t)=\frac{\theta_{11}(t)\times \theta_{22}(t)-\theta_{21}(t)\times \theta_{12}(t)}{\sqrt{\left[\theta_{11}(t)+\theta_{21}(t)]\times [\theta_{11}(t)+\theta_{12}(t)]\times [\theta_{22}(t)+\theta_{21}(t)]\times [\theta_{22}(t)+\theta_{12}(t)\right]}},$$

and *κ*_7_ is the Fowlkes–Mallows index (FMI).

(19) $$\kappa_{7}(t)=\sqrt{\frac{\theta_{11}(t)}{\theta_{11}(t)+\theta_{21}(t)}\times \frac{\theta_{11}(t)}{\theta_{11}(t)+\theta_{21}(t)}}$$

There are two indicators *κ*_4_ and *κ*_6_ using all the four basic measures (*θ*_11_, *θ*_12_, *θ*_21_, *θ*_22_). This study finally chooses *κ*_6_ as the most important indicator due to its larger range (–1 ≤ *κ*_6_ ≤ +1) than that of *κ*_4_ (0 ≤ *κ*_4_ ≤ 1).

The previous process is for one run of *I*-fold CV. The experiment runs the *I*-fold CV *T* runs. After all runs, the mean and standard deviation (MSD) of all seven indicators $\vec{\kappa }=\{\kappa_{m}(m=1,\ldots ,7)\}$ are gauged over *T* runs.

(20) $$\left\{ \begin{array}{l} \mu (\kappa_{m})=\frac{1}{T}\times \sum_{t=1}^{T}\kappa_{m}(t)\\ \sigma (\tau_{m})=\sqrt{\frac{1}{T-1}\times \sum_{t=1}^{T}|\kappa_{m}(t)-\mu (\kappa_{m}){|}^{2}} \end{array}\right.,m=1,\ldots ,7,$$

where *μ* signifies the mean value and *σ* the standard deviation. The values of MSD are recorded in the format of *μ* ± *σ*.

## 4 Experiments, Results, and Discussions

### 4.1 Hyperparameter Setting

The hyperparameters are listed in Table 3. The minimum and maximum gray values of HSed images are (0, 255). The size of the downsampled image is 227 × 227. We have in total *b*_1_ = 9 different DA methods on both raw image and HMI. Every DA produces *b*_2_ = 30 images. The AFMDA is *b*_3_ = 542. Activation function in ELM is chosen the sigmoid function. The number of hidden neurons in ELM is set to *L* = 2000. We run ten runs of 10-fold CV to report the robust results.

### 4.2 Results of MDA

**Figure 7:**
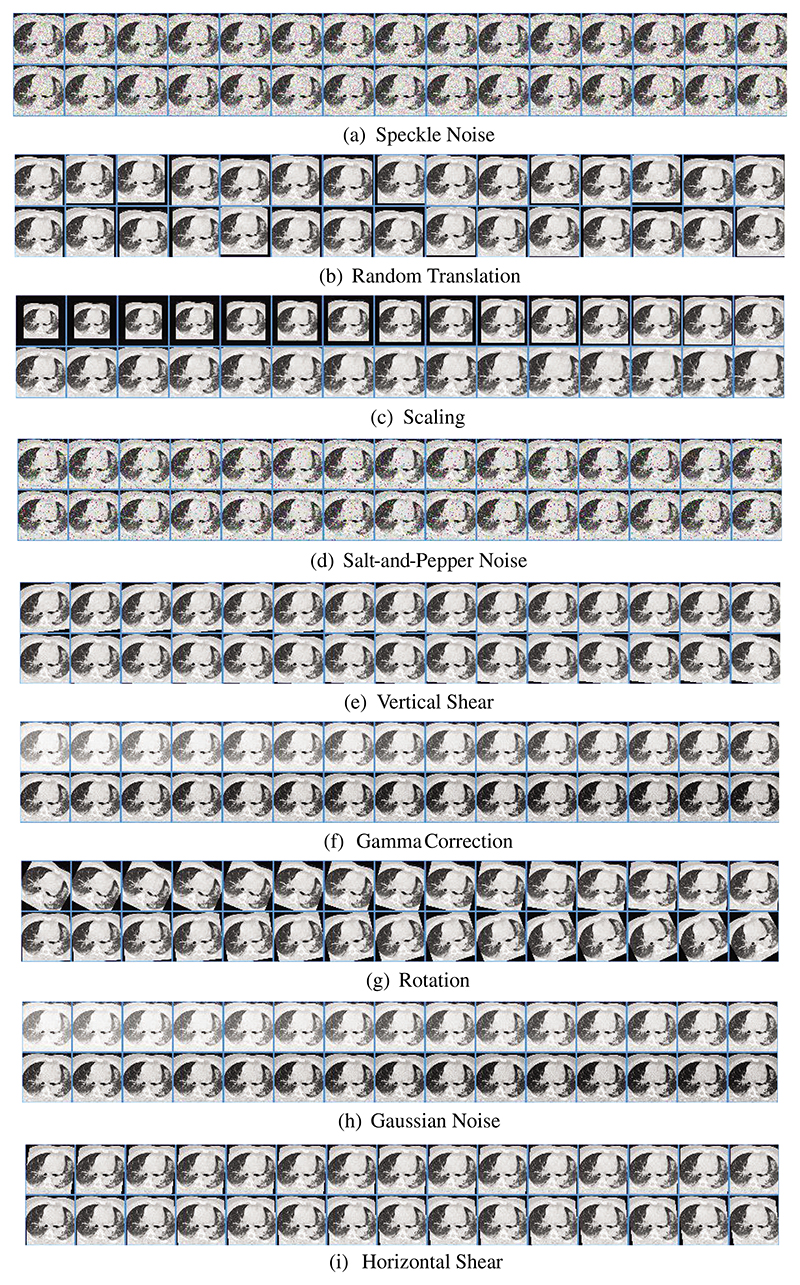
Result of MDA

The MDA result of Fig. 1c is shown in Fig. 7, in which we can observe the nine DA results, i.e., speckle noise, random translation, scaling, salt-and-pepper noise, vertical shear, Gamma correction, rotation, Gaussian noise, and horizontal shear. Due to the space limit, the nine DA outcomes on HMI are not displayed. Fig. 7 indicates that the MDA can increase the diversity of the training set.

Meanwhile, the AFMDA value *b*_3_ = 542 makes the training burden of our model 542 times as much as that of the model without MDA. Nevertheless, in the test stage, there is no need to apply MDA to the test images, so our model is the same quick as the model without MDA.

### 4.3 Results of Proposed SNELM Model

Table 4 displays the ten runs of 10-fold CV, where *t* = 1, 2, ... , 10 means the run index. For the dataset D1, SNELM attains a sensitivity of 96.35 ± 1.50%, a specificity of 96.08 ± 1.05%, a precision of 96.10 ± 1.00%, an accuracy of 96.22 ± 0.94%, an F1 score of 96.22 ± 0.95%, an MCC of 92.45 ± 1.87%, and an FMI of 96.22 ± 0.95%. For the dataset D2, SNELM attains a sensitivity of 96.00 ± 1.25%, a specificity of 96.28 ± 1.16%, a precision of 96.28 ± 1.13%, an accuracy of 96.14 ± 0.96%, an F1 score of 96.13 ± 0.96%, an MCC of 92.29 ± 1.91%, and an FMI of 96.14 ± 0.96%.

### 4.4 Confusion Matrix and ROC Curve

**Figure 8:**
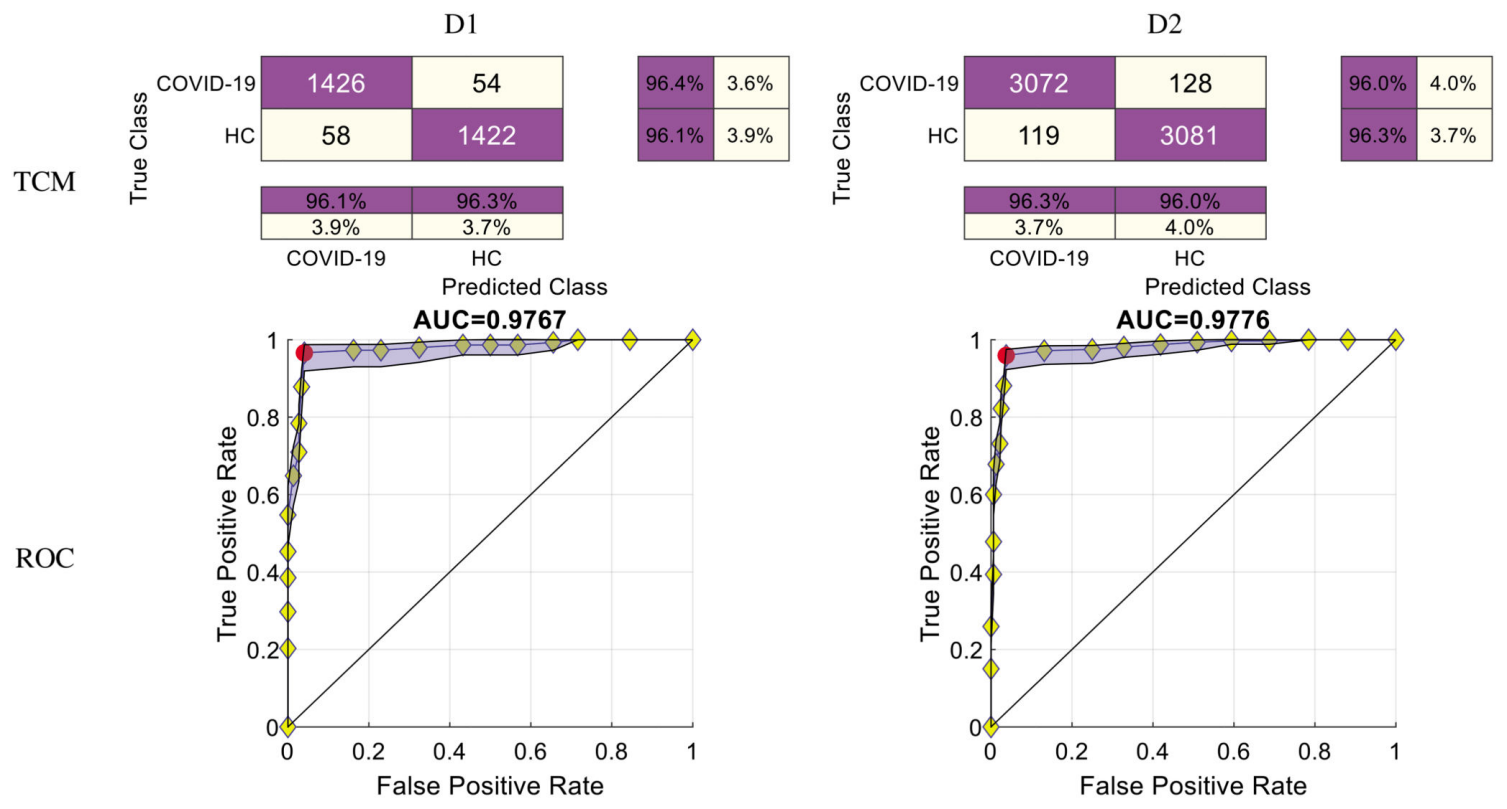
TCMs and ROC curves of two datasets

After combining the ten runs altogether, we can draw the overall TCMs and the ROC curves of the two datasets. The top row of Fig. 8 displays the TCM of two datasets. The bottom row of Fig. 8 displays their corresponding ROC curves. The AUC values of D1 and D2 are 0.9767 and 0.9776, respectively.

### 4.5 Comparison with State-of-the-Art Models

The SNELM model is compared with seven state-of-the-art COVID-19 recognition models over two datasets. The comparison models consist of FSV [3], 3SBBO [4], CNNSP [5], GCMSVM [6], WEJ [7], GCMSNN [8], SaPSO [9], DLA [10], DeCovNet [11], and DLM [12]. Particularly, CNNSP [5], DLA [10], DeCovNet [11], and DLM [12] are deep learning models. The results on two datasets are itemized in Table 5. As we can observe, the proposed SNELM outperforms other state-of-the-art models in both datasets.

**Figure 9:**
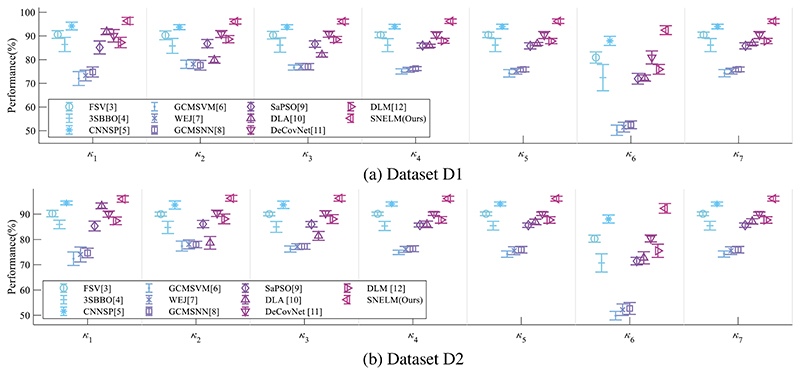
EBs of model comparison

Error bar (EB) can assist in observing the differences in the model’s performances. Fig. 9 displays the EB of different models over two datasets. It shows that the performance of this proposed SNELM model is higher than those of seven state-of-the-art models. The reason of the success of SNELM model may lie in three points: (i) MDA helps increase the size of training set significantly. (ii) The SN with complex bypass helps extract efficient features. (iii) ELM serves as an effective classifier.

## 5 Conclusions

This study proposes an innovative SNELM model for COVID-19 detection. The MDA is used to increase the size of the training set. The SN with complex bypass is employed to generate SN features. ELM is used as the classifier. This proposed SNELM model can produce higher results than seven state-of-the-art models.

There are three deficiencies of the proposed SNELM model: (i) Strict clinical validation is not tested. (ii) The SNELM model is a black box. (iii) Other chest-related infectious diseases are not considered.

In our future studies, our team first shall distribute the proposed SNELM model to the online cloud computing environment (such as Microsoft Azure or Amazon Web Services). Second, we intend to incorporate Gram-CAM into this model to make it explainable. Third, chest-related infectious diseases, such as tuberculosis or pneumonia, will be added to our task.

## Figures and Tables

**Table 1:** Two COVID-19 datasets

Dataset	*n*_1_	*n*_2_
D1 [4]	66 + 66	148 + 148
D2 [5]	142 + 142	320 + 320

**Table 2:** Abbreviation and meaning

Abbreviation	Meaning
AFMDA	Augmentation factor of MDA
CCT	Chest computed tomography
CNN	Convolutional neural network
CV	Cross-validation
EL	Expand layer
ELM	Extreme learning machine
FMI	Fowlkes–Mallows index
HMI	Horizontally mirrored image
HS	Histogram stretching
MCC	Matthews correlation coefficient
MDA	Multiple-way data augmentation
MSD	Mean and standard deviation
MTC	Margin and text crop
PCR	Polymerase chain reaction
SL	Squeeze layer
SN	SqueezeNet
TCM	Test confusion matrix

**Table 3:** Hyperparameter setting

Parameter	Value
(*u*_min_, *u*_max_)	(0, 255)
(*a*_1_, *a*_2_)	(227, 227)
*b*_1_	9
*b*_2_	30
*b*_3_	542
*h*	sigmoid
*L*	2000
*I*	10
*T*	10

**Table 4:** Results often-run 10-fold CV of the proposed SNELM model

Dataset	*t*	*κ*_1_	*κ*_2_	*κ*_3_	*κ*_4_	*κ*_5_	*κ*_6_	*κ*_7_
D1	1	97.30	94.59	94.74	95.95	96.00	91.93	96.01
	2	94.59	96.62	96.55	95.61	95.56	91.23	95.57
	3	97.97	95.95	96.03	96.96	96.99	93.94	96.99
	4	97.30	94.59	94.74	95.95	96.00	91.93	96.01
	5	94.59	96.62	96.55	95.61	95.56	91.23	95.57
	6	95.95	95.95	95.95	95.95	95.95	91.89	95.95
	7	93.92	95.27	95.21	94.59	94.56	89.20	94.56
	8	96.62	96.62	96.62	96.62	96.62	93.24	96.62
	9	97.30	96.62	96.64	96.96	96.97	93.92	96.97
	10	97.97	97.97	97.97	97.97	97.97	95.95	97.97
	MSD	96.35 ± 1.50	96.08 ± 1.05	96.10 ± 1.00	96.22 ± 0.94	96.22 ± 0.95	92.45 ± 1.87	96.22 ± 0.95
D2	1	94.38	96.56	96.49	95.47	95.42	90.96	95.42
	2	97.50	97.50	97.50	97.50	97.50	95.00	97.50
	3	95.62	95.62	95.62	95.62	95.62	91.25	95.62
	4	96.25	95.00	95.06	95.62	95.65	91.26	95.65
	5	97.19	97.19	97.19	97.19	97.19	94.38	97.19
	6	97.50	98.12	98.11	97.81	97.81	95.63	97.81
	7	96.88	94.69	94.80	95.78	95.83	91.58	95.83
	8	95.62	95.00	95.03	95.31	95.33	90.63	95.33
	9	94.69	96.56	96.50	95.62	95.58	91.27	95.59
	10	94.38	96.56	96.49	95.47	95.42	90.96	95.42
	MSD	96.00 ± 1.25	96.28 ± 1.16	96.28 ± 1.13	96.14 ± 0.96	96.13 ± 0.96	92.29 ± 1.91	96.14 ± 0.96

**Table 5:** Comparison of the proposed SNELM with SOTA models (Unit: %)

Dataset	Model	*κ*_1_	*κ*_2_	*κ*_3_	*κ*_4_	*κ*_5_	*κ*_6_	*κ*_7_
D1	FSV [3]	90.61 ± 1.64	90.27 ± 1.86	90.33 ± 1.62	90.44 ± 1.19	90.46 ± 1.17	80.90 ± 2.37	90.46 ± 1.17
	3SBBO [4]	86.40 ± 3.00	85.81 ± 3.14	86.14 ± 3.03	86.12 ± 2.75	86.16 ± 2.77	72.42 ± 5.55	86.15 ± 2.76
	CNNSP [5]	94.19 ± 1.63	93.72 ± 1.06	93.75 ± 0.97	93.95 ± 0.96	93.96 ± 0.99	87.92 ± 1.92	93.97 ± 0.98
	GCMSVM [6]	72.03 ± 2.94	78.04 ± 1.72	76.66 ± 1.07	75.03 ± 1.12	74.24 ± 1.57	50.20 ± 2.17	74.29 ± 1.53
	WEJ [7]	73.31 ± 2.26	78.11 ± 1.92	77.03 ± 1.35	75.71 ± 1.04	75.10 ± 1.23	51.51 ± 2.07	75.14 ± 1.22
	GCMSNN [8]	74.80 ± 2.11	77.64 ± 2.05	77.02 ± 1.34	76.22 ± 0.83	75.86 ± 1.00	52.49 ± 1.64	75.89 ± 0.98
	SaPSO [9]	85.14 ± 2.74	86.76 ± 1.75	86.57 ± 1.36	85.95 ± 1.14	85.82 ± 1.30	71.95 ± 2.26	85.83 ± 1.30
	DLA [10]	91.82 ± 1.25	79.86 ± 1.38	82.03 ± 0.93	85.84 ± 0.65	86.64 ± 0.61	72.23 ± 1.30	86.78 ± 0.62
	DeCovNet [11]	90.07 ± 2.63	90.81 ± 1.47	90.76 ± 1.32	90.44 ± 1.39	90.39 ± 1.49	80.92 ± 2.75	90.40 ± 1.48
	DLM [12]	87.23 ± 2.19	88.65 ± 1.52	88.51 ± 1.27	87.94 ± 1.03	87.84 ± 1.11	75.92 ± 2.06	87.86 ± 1.11
	SNELM (Ours)	**96.35 ± 1.50**	**96.08 ± 1.05**	**96.10 ± 1.00**	**96.22 ± 0.94**	**96.22 ± 0.95**	**92.45 ± 1.87**	**96.22 ± 0.95**
D2	FSV [3]	90.25 ± 1.27	90.03 ± 0.80	90.06 ± 0.72	90.14 ± 0.70	90.15 ± 0.73	80.29 ± 1.41	90.15 ± 0.74
	3SBBO [4]	85.94 ± 1.68	84.75 ± 2.42	84.96 ± 2.16	85.34 ± 1.81	85.44 ± 1.74	70.71 ± 3.61	85.44 ± 1.73
	CNNSP [5]	94.44 ± 0.73	93.63 ± 1.60	93.70 ± 1.47	94.03 ± 0.80	94.06 ± 0.76	88.08 ± 1.59	94.05 ± 0.75
	GCMSVM [6]	72.38 ± 2.68	77.38 ± 1.96	76.22 ± 1.21	74.88 ± 0.86	74.21 ± 1.25	49.85 ± 1.70	74.25 ± 1.21
	WEJ [7]	74.06 ± 2.96	78.06 ± 1.81	77.17 ± 1.17	76.06 ± 1.18	75.55 ± 1.58	52.21 ± 2.28	75.58 ± 1.54
	GCMSNN [8]	74.66 ± 1.87	78.00 ± 1.29	77.24 ± 1.15	76.33 ± 1.18	75.92 ± 1.31	52.70 ± 2.34	75.93 ± 1.30
	SaPSO [9]	85.31 ± 1.94	86.09 ± 1.43	86.01 ± 1.10	85.70 ± 0.76	85.64 ± 0.87	71.44 ± 1.49	85.65 ± 0.86
	DLA [10]	93.28 ± 1.14	78.66 ± 2.51	81.41 ± 1.76	85.97 ± 1.29	86.93 ± 1.10	72.74 ± 2.41	87.14 ± 1.06
	DeCovNet [11]	90.03 ± 1.22	90.34 ± 1.25	90.33 ± 1.07	90.19 ± 0.68	90.17 ± 0.69	80.39 ± 1.35	90.18 ± 0.68
	DLM [12]	87.37 ± 1.51	88.12 ± 1.94	88.06 ± 1.75	87.75 ± 1.31	87.71 ± 1.29	75.52 ± 2.62	87.71 ± 1.29
	SNELM (Ours)	96.00 ± 1.25	96.28 ± 1.16	96.28 ± 1.13	96.14 ± 0.96	96.13 ± 0.96	92.29 ± 1.91	96.14 ± 0.96

Note: Bold means the best. CNNSP [5], DLA [10], DeCovNet [11], and DLM [12] are deep learning models.
